# Do Cognitive Subtypes Exist in People at Clinical High Risk for Psychosis? Results From the EU-GEI Study

**DOI:** 10.1093/schbul/sbae133

**Published:** 2024-07-25

**Authors:** George Gifford, Alessia Avila, Matthew J Kempton, Paolo Fusar-Poli, Robert A McCutcheon, Fiona Coutts, Stefania Tognin, Lucia Valmaggia, Lieuwe de Haan, Mark van der Gaag, Barnaby Nelson, Christos Pantelis, Anita Riecher-Rössler, Rodrigo Bressan, Neus Barrantes-Vidal, Marie-Odile Krebs, Birte Glenthøj, Stephan Ruhrmann, Gabriele Sachs, Bart P F Rutten, Jim van Os, EU-GEI High Risk Study, Philip McGuire

**Affiliations:** Department of Psychiatry, University of Oxford, Oxford, UK; Department of Psychosis Studies, Institute of Psychiatry, Psychology & Neuroscience, King’s College London, London, UK; Faculdade de Medicina, Universidade de Lisboa, Lisbon, Portugal; Faculty of Medicine, Universidade Católica de Lisboa, Lisbon, Portugal; Department of Psychosis Studies, Institute of Psychiatry, Psychology & Neuroscience, King’s College London, London, UK; Department of Psychosis Studies, Institute of Psychiatry, Psychology & Neuroscience, King’s College London, London, UK; Department of Brain and Behavioral Sciences, University of Pavia, Pavia, Italy; Outreach and Support in South-London (OASIS) Service, South London and Maudlsey (SLaM) NHS Foundation Trust, London, UK; Department of Psychiatry and Psychotherapy, University Hospital, Ludwig-Maximilian-University (LMU), Munich, Germany; Department of Psychiatry, University of Oxford, Oxford, UK; Department of Psychosis Studies, Institute of Psychiatry, Psychology & Neuroscience, King’s College London, London, UK; Department of Psychosis Studies, Institute of Psychiatry, Psychology & Neuroscience, King’s College London, London, UK; Department of Psychology, Institute of Psychiatry, Psychology & Neuroscience, King’s College London, London, UK; Department Early Psychosis, AMC, Academic Psychiatric Centre, Amsterdam, The Netherlands; Department of Clinical Psychology, Faculty of Behavioural and Movement Sciences, VU University, Amsterdam, The Netherlands; EMGO+ Institute for Health and Care Research, VU University, Amsterdam, The Netherlands; Parnassia Psychiatric Institute, Department of Psychosis Research, The Hague, The Netherlands; Orygen, Victoria, Melbourne, Australia; Centre for Youth Mental Health, The University of Melbourne, Parkville, Victoria, Australia; Melbourne Neuropsychiatry Centre, University of Melbourne & Melbourne Health, Carlton South, Vic, Australia; Medical Faculty, University of Basel, Basel, Switzerland; Department of Psychiatry, Interdisciplinary Lab for Clinical Neurosciences (LiNC), Universidade Federal de Sao Paulo (UNIFESP), Sao Paulo, Brazil; Departamento de Psicologia Clínica i de la Salut (Universitat Autònoma de Barcelona), Fundació Sanitària Sant Pere Claver (Spain), Spanish Mental Health Research Network (CIBERSAM), Barcelona, Spain; University Paris Descartes, Hôpital Sainte-Anne, C’JAAD, Service Hospitalo-Universitaire, Inserm U894, Institut de Psychiatrie (CNRS 3557), Paris, France; Centre for Neuropsychiatric Schizophrenia Research (CNSR) & Centre for Clinical Intervention and Neuropsychiatric SchizophreSnia Research (CINS), Mental Health Centre Glostrup, University of Copenhagen, Glostrup, Denmark; Faculty of Health and Medical Sciences, Department of Clinical Medicine, University of Copenhagen, Copenhagen, Denmark; Department of Psychiatry and Psychotherapy, University of Cologne, Cologne, Germany; Department of Psychiatry and Psychotherapy, Medical University of Vienna, Vienna, Austria; Department of Psychiatry and Neuropsychology, School for Mental Health and Neuroscience, Maastricht University Medical Centre, Maastricht, The Netherlands; Department of Psychiatry and Neuropsychology, School for Mental Health and Neuroscience, Maastricht University Medical Centre, Maastricht, The Netherlands; Department of Psychiatry, University of Oxford, Oxford, UK; Department of Psychiatry, University of Oxford, Oxford, UK; Department of Psychosis Studies, Institute of Psychiatry, Psychology & Neuroscience, King’s College London, London, UK

**Keywords:** clinical high risk for psychosis, cognition, unsupervised learning, clustering

## Abstract

**Background and Hypothesis:**

Cognition has been associated with socio-occupational functioning in individuals at Clinical High Risk for Psychosis (CHR-P). The present study hypothesized that clustering CHR-P participants based on cognitive data could reveal clinically meaningful subtypes.

**Study Design:**

A cohort of 291 CHR-P subjects was recruited through the multicentre EU-GEI high-risk study. We explored whether an underlying cluster structure was present in the cognition data. Clustering of cognition data was performed using k-means clustering and density-based spatial clustering of applications with noise. Cognitive subtypes were validated by comparing differences in functioning, psychosis symptoms, transition outcome, and grey matter volume between clusters. Network analysis was used to further examine relationships between cognition scores and clinical symptoms.

**Study Results:**

No underlying cluster structure was found in the cognitive data. K-means clustering produced “spared” and “impaired” cognition clusters similar to those reported in previous studies. However, these clusters were not associated with differences in functioning, symptomatology, outcome, or grey matter volume. Network analysis identified cognition and symptoms/functioning measures that formed separate subnetworks of associations.

**Conclusions:**

Stratifying patients according to cognitive performance has the potential to inform clinical care. However, we did not find evidence of cognitive clusters in this CHR-P sample. We suggest that care needs to be taken in inferring the existence of distinct cognitive subtypes from unsupervised learning studies. Future research in CHR-P samples could explore the existence of cognitive subtypes across a wider range of cognitive domains.

## Introduction

Cognitive impairment is a fundamental component of psychosis.^1–5^ However, the severity of cognitive deficits varies between people with psychosis, which has led researchers to use unsupervised learning methods to search for subtypes of patients with different levels of cognitive ability. This approach has been applied to samples of people with schizophrenia,^6–9^ schizophrenia and bipolar disorder,^10–14^ and with first-episode psychosis (FEP).^15–19^ Although such studies have reported different numbers of cognitive subtypes, all have identified at least one group of patients with relatively poor cognition and one group with unimpaired cognition across cognitive domains.

Given strong evidence for cognitive deficit in Clinical High Risk for Psychosis (CHR-P),^1,20–22^ several studies have used cognition data to cluster CHR-P samples.^23–25^ One study identified a four-cluster solution in a sample of CHR-P, family history of psychosis, and HC participants, with the low-cognition subtype showing higher risk of transition to psychosis and worse baseline and follow-up functioning.^23^ Several studies have reported two-cluster solutions of “spared” and “impaired” cognition clusters: one in a mixed sample of HC, CHR-P, and FEP participants, reporting lower functioning in the low-cognition subtype but no difference in CHR-P symptomatology or transition rate,^24^ and another study in a mixed sample of recent onset depression, FEP, and CHR-P participants, reporting no difference in symptomatology or functioning between cognitive subtypes in the CHR-P group.^25^

Validation of clustering results in psychosis populations has typically relied on the presence of clinically or biologically meaningful differences between clusters. For instance, studies have shown lower cognitive ability subtypes to have lower levels of functioning^7,9,12,19^ and poorer functional outcomes.^6,12,18^ Additionally, some studies have identified differences between cognitive subtypes in negative symptomatology^7,9,16–19^ and measures of brain volume.^8,19,26^

Problematically, many commonly used clustering algorithms such as K-means clustering will generate a clustering solution, regardless of whether a clear underlying cluster structure exists in the data. In these cases, comparing differences in secondary variables, such as measures of functioning and symptomatology, could simply reflect an association of that variable with the cognitive data. Ideally, such external validation results should be complemented by appropriate internal validation methods that describe the underlying cluster structure.

The present study aimed to extend the existing literature by performing a clustering analysis of cognition data in a large sample of CHR-P individuals. We sought to substantiate clustering solutions, first by assessing the presence of an underlying cluster structure using internal validation and visualization techniques, and then by comparing measures of functioning, psychosis symptoms, transition outcome, and grey matter volume between subtypes. Grey matter volume has been used in previous studies to validate cognitive clustering results in samples of participants with psychosis^8,19,26^ and there is evidence that it can be used as a marker of CHR-P status.^27,28^ We considered differences in negative and basic symptoms between cognitive subtypes. Negative symptoms include features which may affect cognitive performance, such as amotivation and alogia, and negative symptoms have been associated with cognitive impairment in CHR-P.^29–32^ Basic symptoms comprise a set of subjective cognitive disturbances that are evident in CHR-P subjects,^33^ including symptoms such as thought block, disturbance of expressive speech, and an inability to divide attention.

We tested the following hypotheses: (1) Cognitive data would exhibit an underlying cluster structure within the CHR-P sample. (2) Cognitive clustering would produce clusters with distinct levels of cognitive performance. (3) Cognitive clusters would differ in terms of functioning, severity of basic and negative symptoms, regional grey matter volume, and the subsequent incidence of psychosis.

## Methods

### Participants

Participants were recruited through the EU-GEI High-Risk study. CHR-P status was defined using the Comprehensive Assessment of At-Risk Mental States (CAARMS) criteria.^34^ All participants had no history of psychotic disorder, neurological disorder or drug/alcohol dependency that would explain relevant CHR-P symptoms, contraindications for Magnetic Resonance Imaging (MRI), or an IQ estimate <60 according to the shortened WAIS-III protocol.^35^ Healthy controls (HC) were included if they did not meet CAARMS criteria. Written informed consent was obtained for every participant and the study was conducted in accordance with the Declaration of Helsinki. Recruitment procedures and inclusion criteria have been described in detail in previous studies.^36–38^

The initial sample included 344 CHR-P and 67 HC participants. Participants were removed from the study if they had more than 20% missing data across relevant variables (see procedures for measures) (CHR-P N = 53, HC N = 10). Demographic, symptom, and functioning differences were compared between included and excluded participants to check for bias. The final sample included 291 CHR-P participants and 53 controls. Scores from HC participants were used to determine the severity of impairment of the CHR-P sample.

### Procedures

Participants in the EU-GEI study were assessed with a range of clinical, cognitive, and biological measurements at baseline and follow-up.^39^ Symptom measures used in the current study included the CAARMS,^40^ the Scale for the Assessment of Negative Symptoms (SANS),^41^ and the Schizophrenia Proneness Instrument-Adult version (SPI-A) to assess basic symptoms.^33^ The Global Assessment of Functioning Disability (GAF-D) scale^42^ was used to measure socio-occupational functioning. Symptom, functioning, transition to psychosis, and grey matter volume were used to explore the validity of clustering solutions. Transition to a full psychotic disorder was determined using the structured clinical interview for DSM-IV-TR axis I^43^ and CAARMS.^40^ Cognitive tasks used in the current study are summarized in table 1. In the present study, cognitive variables were chosen to cover as wide a range of domains as possible, and included processing speed, attention, working memory, verbal learning, reasoning, social cognition, verbal fluency, general knowledge, and visual-perceptual organization.

**Table 1.** Cognitive Tasks and Associated Cognitive Domains Used as Features for Unsupervised Learning in the Current Study

Cognitive Task	Domain
Trail-making task part A^44^	Processing speed, visual attention
Digit symbol coding^45^	Processing speed, working memory, visuospatial processing
Arithmetic^45^	Mathematical reasoning, working memory
Block design^45^	Visual-perceptual organization
Information^45^	General knowledge
Digit span forwards/backwards^45^	Cognitive control, working memory
BEADS task^46^	Reasoning
Benton facial recognition^47^	Social cognition
Rey auditory verbal learning task^48^	Immediate/delayed verbal recall
Verbal fluency test^49^	Semantic/phonemic fluency

### Data Preprocessing

Data preprocessing and analysis were performed in R v4.1.^50^ For the clustering and network analysis the following preprocessing steps were taken: (1) Cognition and symptom score data were imputed using multiple imputation by chained equations.^51^ Mean % of missing data across variables = 2.37% (SD = 2.28%; max = 7.9%). (2) Age, sex, and years of education statistical effects were removed using linear regression. (3) Site effects were removed using ComBat^52^ using the SVA package.^53^

### Clustering and Cluster Validation

K-means clustering was performed on 13 preprocessed cognitive features. The optimal number of clusters was judged based on the elbow criterion of silhouette scores. K-means clustering will assign cluster labels regardless of an underlying cluster structure, therefore density-based spatial clustering of applications with noise (DBSCAN) was also performed because it infers the number of clusters from the data. DBSCAN was run using a range of minimum data points (5–30 in steps of 5) and over a range of Epsilon values (ε = 1–5). Density-based clustering validation (DBCV) scores were used to find the optimal hyperparameters and to judge cluster quality.^54^

To test the robustness of the results, cluster stability was measured using the Clusterboot R package. We used a subsetting scheme (1000 subsets of 80% of the sample) and a noise scheme (1000 runs), with a mean Jaccard coefficient ≥0.75 indicating a stable cluster.^55^

To further explore whether an underlying cluster structure was present, t-distributed stochastic neighbor embedding (t-SNE) was performed, which allows for visualization of high-dimensionality data in a low-dimensionality space. This has the additional benefit of nonlinear separation of data. t-SNE was performed over multiple perplexity values (10–60), which controls the relative contribution between global and local structures. The underlying covariance structure in the data was additionally explored using principal component analysis (PCA), allowing for visual inspection of directions of maximal variance.

Cluster validity was explored by comparing cognition, functioning (GAF-D scores), symptom (SANS and SPI-A scores), and demographic scores between clusters using *t* tests/Chi-squared tests. Cohen’s D effect sizes (d) were reported for group mean comparisons. Multiple comparisons correction (FDR) was performed over domains with multiple items (cognitive, negative, basic symptoms).

### Structural MRI Preprocessing and Analysis

In a subset of CHR-P subjects (*N* = 201), T1 images were acquired using 3-Tesla MRI scanners.^39^ Acquisition parameters for each site are shown in Supplementary table 1. Demographics were compared between CHR-P subjects with/without sMRI data to screen for selection bias. Grey matter volume maps were computed from sMRI scans using the standard segmentation pipeline in CAT12.8.2^56^ and SPM12 (Wellcome Department of Cognitive Neurology, London, UK; http://www.fil.ion.ucl.ac.uk/spm/software/spm12/). Structural images were checked visually and problematic images were excluded (*N* = 3).^38^ Data with weighted overall image quality scores <3 were further inspected for quality and removed from the analysis if problematic (*N* = 4). This resulted in a subsample of 194 subjects. Grey matter volume maps were smoothed at 8 mm Full-width half maximum and corrected for site differences using the neuroComat Python package. Voxel-based morphometry (VBM) was then used to compare cognition groups, in order to validate clustering solutions. Results were thresholded at p(FDR) ≤ 0.05.

### Network Analysis

To explore the relationship between cognition, symptomatology, and functioning in this sample, associations between variables were visualized as a network. This approach has the benefit of clearly representing each unifactorial association between pairs of scores, while allowing for an exploration of “network community structure,” which describes the clustering of network nodes based on their level of association.^57^ The network was formed using Spearman’s correlations, with negative correlations being discarded. Cognition scores were reversed so that worse performance would correlate positively with higher symptom scores. Nodes were removed if their degree <1. A Louvain clustering algorithm^58^ (resolution γ = 0.5) was used to suggest the grouping of items into separate communities. This was repeated at multiple resolutions (γ range = 0.25, 0.5, 1.0, 1.25) to explore the stability of results.

## Results

### Demographics

Demographics are shown in table 2. CHR-P and HC groups were balanced in terms of age (t (DF) = −1.23 (81.09), *P* = .222) and gender (x2 = 0.00, *P* = .999). The CHR-P group had lower years of education, estimated IQ, and functioning scores (table 2). The mean follow-up period for CHR-P was 643 days (SD = 255.41). The mean number of days to transition was 378.29 (SD = 1.16) and the maximum number of days to transition was 2220 days (6.08 years). Included and excluded CHR-P participants did not differ in terms of age (t (DF) = 1.8 (78.61), *P* = .076), gender (x2 = 0.00, *P* = .999), years of education (t (DF) = −0.34 (43.52), *P* = .736), GAF disability score (t (DF) = −0.73 (59.14), *P* = .47), or estimated IQ (t (DF) = −0.65 (29.57), *P* = .521).

**Table 2.** Demographics (Mean Age, Sex, Mean Years of Education), Mean Estimated IQ, Baseline/Follow-up Mean GAF, Transitioned to Psychosis, Days to Follow Up, Mean CAARMS Positive/Negative Summary Scores, and Baseline Medication Use for the CHR-P and HC Samples

	CHR-P	HC	T/X Stat	*P* Value
*N*	291	53		
Age (SD)	22.58 (4.98)	23.38 (4.20)	−1.23	.222
Sex	157 (53.95%)/134 (46.05%)	28 (52.83%)/25 (47.17%)	0.00	.999
Years of education (SD)	14.35 (3.10)	16.26 (2.73)	−4.55	<.001
Estimated IQ (SD)	98.24 (16.74)	112.34 (18.21)	−5.24	<.001
Basic symptom criteria met	146 (59.11%)			
Genetic vulnerability	44 (16.79%)			
Attenuated symptoms	241 (87.64%)			
BLIP	23 (8.65%)			
GAF symptom (SD)	54.75 (10.30)	87.52 (11.02)	−19.89	<.001
GAF disability (SD)	55.26 (12.38)	86 (8.94)	−21.37	<.001
GAF symptom 2 years (SD)	60.13 (13.60)	86.64 (9.10)	−15.41	<.001
GAF disability 2 years (SD)	61.92 (14.53)	86.31 (6.96)	−16.31	<.001
Transition (SD)	56 (19.24%)			
Days to transition (SD)	378.29 (1.16)			
Days to follow up (SD)	643 (255.41)	692.15 (184.20)	−1.39	.168
CAARMS positive mean (SD)	2.51 (1.01)			
CAARMS negative mean (SD)	1.98 (0.97)			
Antidepressant	80 (29.96%)			
Antipsychotic	26 (9.59%)			

### Clustering Results

**Fig. 1.**
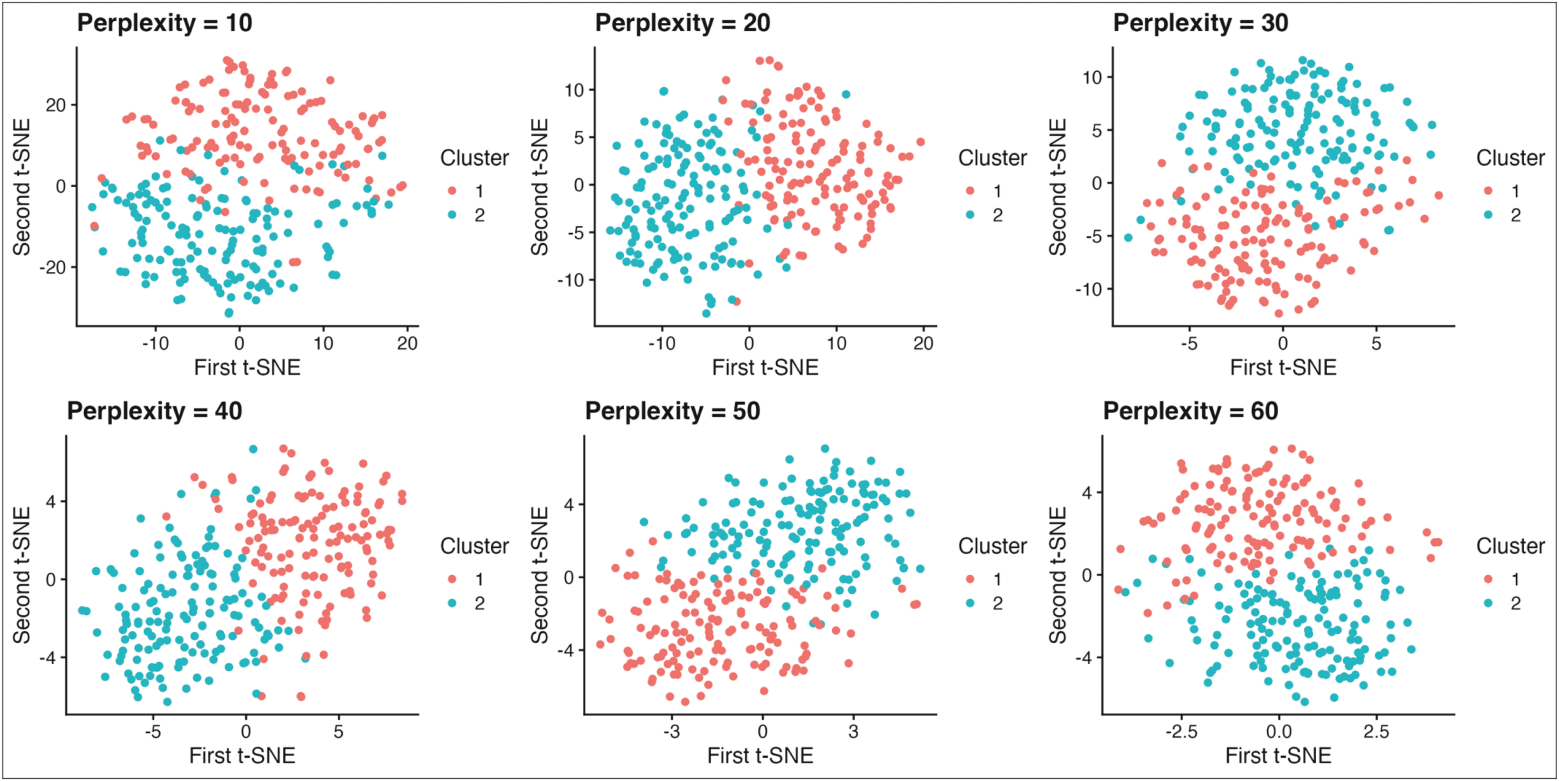
Scatterplots showing first and second t-SNE computed on the cognition data collected in CHR-P participants. Results are reported over perplexity values 10–60. Clusters 1 and 2 computed using k-means clustering of cognition data with k = 2.

**Fig. 2.**
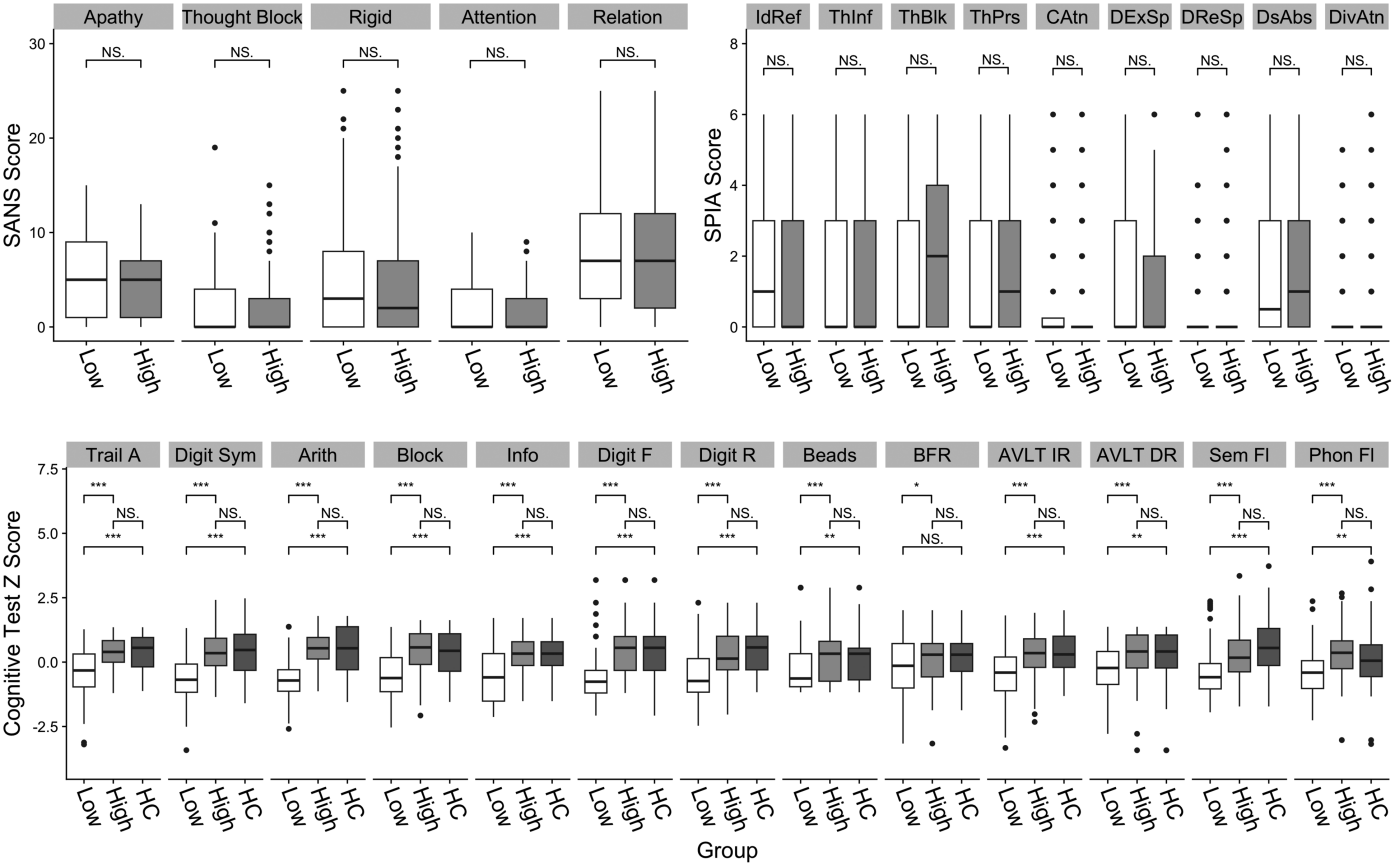
Top panel: SANS and SPIA scores between high/low CHR-P cognition groups defined using k-means clustering (k = 2), and HC samples. IdRef, ideas of reference; ThInf, thought interference; ThBlk, thought block; ThPrs, thought pressure; CAtn, captivation of attention; DExSp, disturbance of expressive speech; DReSp, disturbance of receptive speech; DsAbs, disturbance of abstract thinking; DivAtn, inability to divide attention. Lower panel: Cognition scores between high-/low-CHR-P cognition groups defined using k-means clustering (k = 2), and HC samples. Trail A, Trail-making task part A; Digit Sym, digit symbol; Arith, arithmetic; Block, block design; Info, information; Digit F, digit span task forward; Digit R, digit span task reverse; Beads, beads jumping to conclusions task; BFR, Benton facial recognition task; AVLT IR/DR, auditory verbal learning task immediate recall/delayed recall; Sem Fl, Verbal learning semantic fluency; Phon Fl, verbal learning phonemic fluency (*pFDR ≤ 0.05, **pFDR ≤ 0.01, ***pFDR ≤ 0.001).

For k-means clustering, silhouette scores suggested a two-cluster solution to be optimal (Supplementary figure 1), however the silhouette score was low (0.16) suggesting poorly formed clusters. DBSCAN clustered the data into a maximum of 2 clusters and the DBCV scores^54^ ranged from −0.62 to 0.03 suggesting poor cluster formation. Visual inspection of t-SNE plots did not identify any underlying clusters in the data (figure 1). Cluster stability was however high for both k-means (mean Jaccard Index: subset method = 0.96, 0.96; noise method = 0.95, 0.95) and DBSCAN (mean Jaccard Index: subset method = 0.96, 0.78; noise method = 0.99, 0.93). Despite no clear underlying cluster structure, K-means clustering with k = 2 separated data into one high cognition cluster with cognition scores that did not differ significantly from HCs and one low-cognition cluster that showed significantly lower cognition in all domains except for social cognition (figure 2). As these cognitive clusters were similar to “spared” and “impaired” cognition subtypes reported in previous studies^19,24,25^ the k-means k = 2 clustering solution was used for further validation. Clusters are hereafter referred to as high- and low-cognition subtypes.

There were no differences between high-/low-cognition subtypes in demographics, functioning, or rate of transition to psychosis (Supplementary table 2), and no differences between clusters in levels of negative and basic symptoms (figure 2). In order to explore the possibility that using the dimensional features of cognitive data would be better suited to establishing a relationship between cognition and key outcome variables, we used multiple regression to test for an association between GAF disability scores and cognition predictors, and between transition to psychosis and cognition predictors, while controlling for age, sex, and years of education. In a multiple linear regression model, there was no significant association between cognition and GAF disability scores (F(16, 206) = 1.34, *P* = .174, R2 = 0.02) and in a logistic regression model there was no significant association between cognition and transition to psychosis (x2(16) = 19.50, *P* = .243).

### PCA

PCA applied to the cognition data showed the main principal component to be a general cognition factor (Supplementary figure 2) which accounted for 28.73% of the variance. This general factor was highly correlated with estimated IQ (R = 0.77, *P* < .001), whereas the mean of the other PCA factors (PCA factors 2–13; variance explained = 71.27%) across participants was not (R = 0.11, *P* = .682). K-means clustering (k = 2) using the 10 first principal components (90.41% variance explained) resulted in a highly similar clustering solution (96.56% overlap) as did clustering of the 5 first principal components (64.70% variance explained; 96.91% overlap). However, k-means clustering (k = 2) of components 2–10 (61.68% variance explained) resulted in a highly dissimilar clustering solution (52.92% overlap). This clustering solution did not produce better fitting clusters (silhouette score = 0.11) or separate participants into high/low clusters with different levels of functioning: high (SD) = 55.26 (13.33), low (SD) = 55.27 (11.02), t (DF) = −0.01 (281.98), *P* = .994, d = 0.00.

### Structural MRI

CHR-P participants with/without sMRI data did not differ in terms of demographics, IQ, positive/negative symptom levels, functioning, or medication use (Supplementary table 3). In comparing participants in high-/low-cognition clusters, no differences in grey matter volume were found at p(FWE) < 0.05. Additionally, no differences in grey matter volume were found between high-/low-cognition subtypes defined by a median split of the cognition composite score.

### Network Analysis

**Fig. 3.**
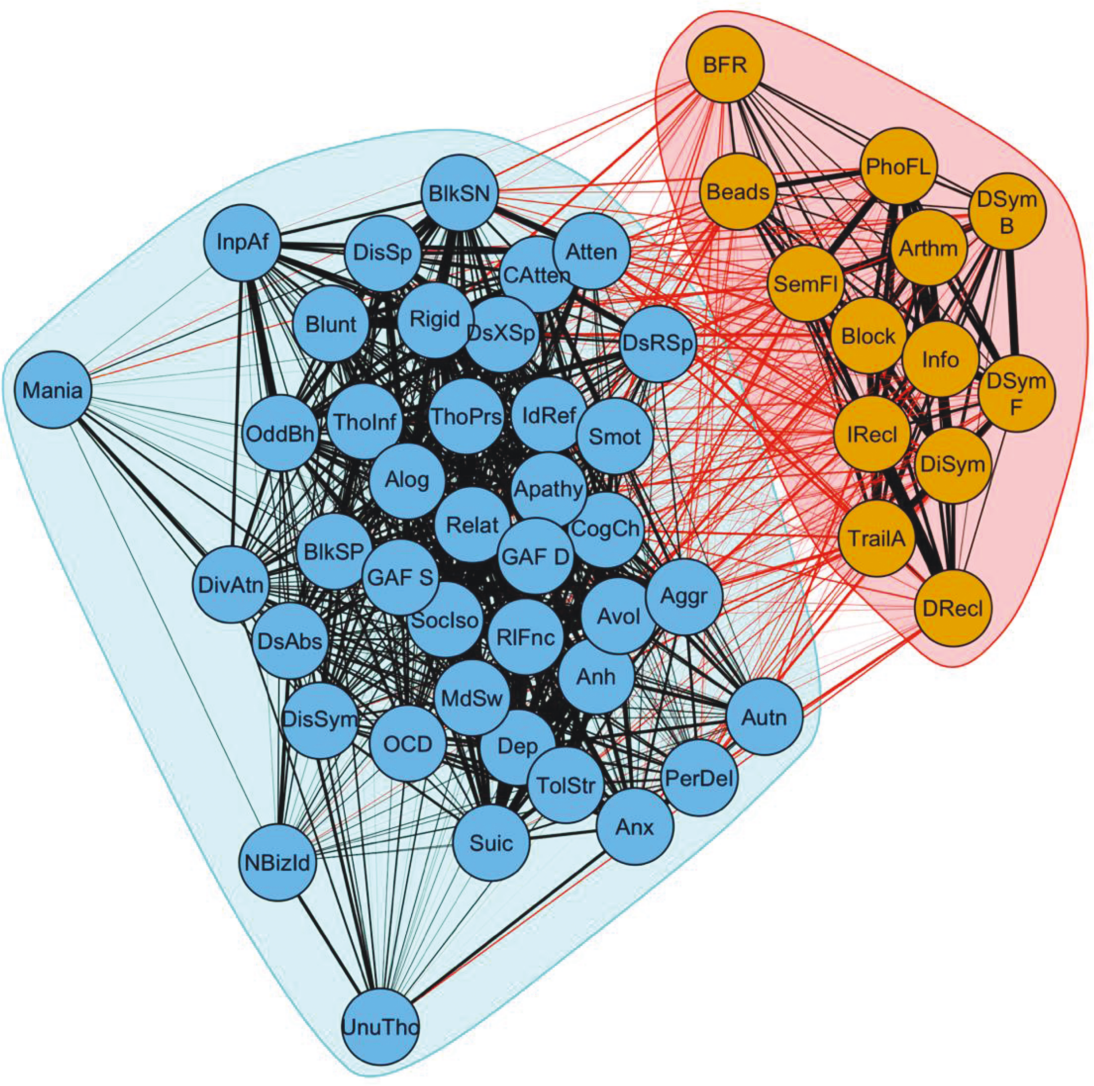
Weighted network of Spearman’s correlations between cognition, functioning, and symptom scores. Nodes with a degree < 1 were removed. Colors indicate communities of nodes defined using a Louvain community detection algorithm (resolution parameter γ = 0.5). Black edges show within community connections, red edges show between community connections. Cognition: TrailA, Trail-making task; DiSym, digit symbol; Arthm, arithmetic; Block, block design; Info, information; DS_F, digit span forward; DS_B, digit span reverse; Beads, beads task; BFR, Benton facial recognition; IRecl, AVLT immediate recall; DRecl, AVLT delayed recall; SemFl, semantic fluency; PhoFL, phonemic fluence. Schizophrenia Proneness Instrument-Adult version (SPI-A): IdRef, ideas reference; ThoInf, thought interferences; BlkSP, thought block; ThoPrs, thought pressure; CAtten, captivation attention; DsXSp, disturbance expressive speech; DsRSp, disturbance receptive speech; DsAbs, disturbance abstract thinking; DivAtn, inability divide attention. Scale for the Assessment of Negative Symptoms: Apathy, apathy; BlkSN, thought block; Rigid, rigidity; Atten, attention; Relat, relation. Comprehensive Assessment of At-Risk Mental States: Dep, depression; Suic, suicidality; Anh, anhedonia; Avol, avolition; Mania, mania; MdSw, mood swing; Aggr, aggression; UnuTho, unusual thought; NBizId, nonbizarre ideas; PerDel, perceptual abnormalities; DisSp, disorganized speech; Anx, anxiety; OCD, obsessive compulsive disorder; DisSym, disorganized symptoms; TolStr, tolerance-to-everyday stress; CogCh, cognitive change; Alog, alogia; Blunt, blunted affect; InpAf, inappropriate affect; SocIso, social isolation; RlFnc, role functioning; OddBh, odd behavior; Smot, subjective motor change; Autn, autonomic functioning.

A network of associations between cognition, symptom, and functioning scores suggested two separate communities of nodes: a functioning/symptom community and a cognition community (figure 3). The separation of cognition and symptom/functioning scores was consistent over community algorithm resolution parameters (Supplementary figure 4).

## Discussion

The current study used unsupervised learning to test whether distinct cognitive subtypes could be found in a large cohort of CHR-P participants (*N* = 291). A strength of this study was the detailed assessment of underlying cluster tendency, which suggested there not be a clear cluster structure to the cognition data. Below we discuss the relevance of this finding to the literature of cognitive clustering studies in psychosis.

### Cognitive Clustering in Psychosis

The key challenge for unsupervised learning is the validation of discovered clusters.^59^ Cognitive clustering studies performed in psychosis populations have typically relied on the significance or magnitude of differences in functioning, symptom, or biological measures between clusters to indicate the validity of results. For instance, comparatively poorer socio-occupational functioning has been shown in lower cognition clusters in CHR-P,^23,24^ FEP,^18,19^ and schizophrenia.^6–8,12^ However, as shown in the current study, clustering algorithms may separate participants into subtypes with high-/low-cognition regardless of whether a clustering solution fits the data well. This means that differences between cognitive clusters may simply reflect an association between a given measure and cognition, rather than the existence of an underlying cluster structure. It should be noted that previous studies in CHR-P samples have either not comprehensively explored the tendency of cognitive data to cluster,^23^ or have clustered data using multiple patient groups,^24,25^ which could provide a cluster structure due to the differences in performance between patient populations. While studies that cluster cognitive data across multiple patient groups^11,13,14,19,24,25,60^ may provide valuable insights into transdiagnostic features of mental illness, care should be taken in inferring the existence of distinct subtypes within patient populations using these results.

It is possible that the lack of an underlying cluster structure in the current study was due to the selection of cognition measures. However, the 10 cognition measures used in the current study covered the domains tested in the measurement and treatment to improve cognition in schizophrenia battery,^61^ with the exception of visual learning. In addition, the present study covered a wider range of cognitive domains than many previous cognitive clustering studies in psychosis.^62^ Another possibility is that clustering was heavily influenced by a general factor, and that clustering reflected general intelligence. PCA did indeed show the first principal component to be a general cognition factor, however this explained 28.73% of variance and clustering with this factor removed did not reveal a strong underlying cluster structure. In addition, cognitive deficit in psychosis is often shown to be nondomain specific.^4^

### Cognition and Attenuated Psychosis Symptoms

We hypothesized that cognitive subtypes would differ in terms of the severity of negative and basic symptoms. Previous cognitive clustering studies of CHR-P samples have found, respectively, no symptom differences between cognition subtypes,^24,25^ and significant differences in negative symptoms between 4 clusters.^23^ Similarly, studies that have examined the relationships between cognition and symptoms in CHR-P samples using nonclustering analysis methods have also produced inconsistent results.^29,30,63,64^

In the present study, we found no differences in the symptoms associated with cognitive subtypes. Given the poor internal validity of the clustering results, a supplementary network analysis of associations between cognitive and symptom variables was performed, which revealed that associations between variables clustered into separate symptom and cognition communities. These results support the existence of separate symptom and cognition dimensions, including symptoms of subjective cognitive disturbance.

### Strengths and Limitations

The present study used a large cohort of CHR-P participants (*N* = 291) and employed careful analysis of the underlying cluster structure of the data. In terms of limitations, sMRI data was only available in a subset of participants, however, there were no apparent demographic, functioning, or IQ differences between those with and without sMRI data (Supplementary table 3). There was a bias toward those with higher IQ in those with follow-up data, meaning it was not valid to make comparisons between clusters using follow-up data. A substantial proportion of the original sample was removed due to missing data (15.03%), possibly introducing bias. This may reflect the use of a lengthy and demanding multimodal assessment protocol. However, included/excluded CHR-P participants did not differ in terms of demographics, functioning, or IQ. Lastly, it is possible that different cognitive measures, or a larger cognitive battery, would produce an underlying cluster structure in the CHR-P population.

### Conclusions and Future Directions

Stratifying CHR-P patients in terms of cognitive function could facilitate a more personalized approach to clinical care. Though unsupervised learning methods are well suited to the stratification of patients, the current study did not suggest there to be a clear underlying cluster structure to cognition data in CHR-P. Given inconsistencies in the methodologies of cognitive clustering studies in psychosis^62^ and the inherent difficulties of validating unsupervised learning results, we suggest care needs to be taken in inferring the existence of distinct cognitive subtypes within patient groups from such studies.

The present study suggests that future precision psychiatry studies should treat cognitive data as dimensional. Unsupervised learning may, however be well suited to transdiagnostic approaches to mental health: in exploring the underlying structure of data across diagnostic categories. Further exploration of cognitive subtypes within different psychosis populations and using a wider range of cognitive domains is warranted.

## Supplementary Material

Supplementary material is available at https://academic.oup.com/schizophreniabulletin/.

sbae133_suppl_Supplementary_Material
