# Supplementary material for: Do Cognitive Subtypes Exist in People at Clinical High Risk for Psychosis? Results From the EU-GEI Study
Source: Schizophr Bull. 2024 Jul 25;51(4):1019–29. doi: 10.1093/schbul/sbae133 (PMC12236347; doi:10.1093/schbul/sbae133)
Supplement: sbae133_suppl_Supplementary_Material [file sbae133_suppl_supplementary_material.docx]

Supplementary Materials

*Table S1. Scanner information and image acquisition parameters at each site in the EU-GEI study. Taken with permission from Tognin et al. (2022).*

|  | **London** | **Netherlands 1** | **Netherlands 2** | **Melbourne** | **Basel** | **Paris** | **Vienna** | **Cologne** | **Copenhagen** |
| --- | --- | --- | --- | --- | --- | --- | --- | --- | --- |
|  | **(02)** | **(04, 06)** | **(04, 06)** | **(24)** | **(21)** | **(31)** | **(20)** | **(22)** | **(26)** |
| **Scanner** | 3T GE SIGNA HDx | 3T Philips Intera | 3T Philips Ingenia | 3T Siemens MAGNETOM TrioTim | 3T Siemens MAGNETOM Verio | 3T Siemens MAGNETOM TrioTim | 3T Siemens MAGNETOM TrioTim | 3T Siemens MAGNETOM TrioTim | 3T Philips Achieva |
| **Site** | Centre for Neuroimaging Sciences, IoPPN | Academic Medical Center, University of Amsterdam | Academic Medical Center, University of Amsterdam | Medical Imaging Department, The Royal Children’s Hospital | Clinics for Radiology and Nuclear Medicine, University Hospital Basel | Hospital de la Pitie Salpetriere | The Vienna General Hospital | Max Planck Institute for Neurological Research | Information not available |
| **Head Coil** | 8HR BRAIN MRI | Philips 8 Channel SENSE | Information not available | Siemens 32-channel | Siemens 12-channel | 12-channel | Siemens 12-channel | Siemens Head Matrix 12 channel | 8-channel SENSE |
| **Sequence** | ADNI 2 MPRAGE | ADNI 2 MPRAGE | ADNI 2 MPRAGE | ADNI 2 MPRAGE | ADNI 2 MPRAGE | ADNI 2 MPRAGE | ADNI 2 MPRAGE | ADNI 2 MPRAGE | ADNI 2 MPRAGE |
| **Matrix Size** | 256x256 | 256x240 | 256x240 | 256x240 | 256x240 | 256x240 | 256x240 | 256x240 | 256x240 |
| **Voxel Size (in mm)** | 1.0x1.0x1.2 | 1.0x1.0x1.2 | 1.0x1.0x1.2 | 1.0x1.0x1.2 | 1.0x1.0x1.2 | 1.0x1.0x1.2 | 1.0x1.0x1.2 | 1.0x1.0x1.2 | 1.0x1.0x1.2 |
| **Slice Thick-ness (in mm)** | 1.2 | 1.2 | 1.2 | 1.2 | 1.2 | 1.2 | 1.2 | 1.2 | 1.2 |
| **TR (in ms)** | 6980 | 6800 | 6800 | 2300 | 2300 | 2300 | 2300 | 2300 | 6790 |
| **TE (in ms)** | 2.85 | 3.14 | 3.16 | 2.96 | 2.96 | 2.98 | 2.96 | 2.96 | 3.17 |
| **Flip Angle (°)** | 11 | 9 | 9 | 9 | 9 | 9 | 9 | 9 | 9 |
| **No. of Averages** | 1 | 1 | 1 | 1 | 1 | 1 | 1 | 1 | 1 |
| **Acquisition Time (in min)** | 10 | 9 | 9 | 9 | 9 | 9 | 9 | 9 | 9 |

**Abbreviations:** min, minutes; T, Tesla; TE, echo time; TR, repetition time.

Note: Participants recruited in the Amsterdam and the Hague sites were all scanned in the University of Amsterdam Imaging center. The scanner was changed halfway through the study which is labeled as The Netherlands 1 and The Netherlands 2. Parameters between scanner manufacturers are expected to be different as ADNI adjusted these to harmonize image contrast.


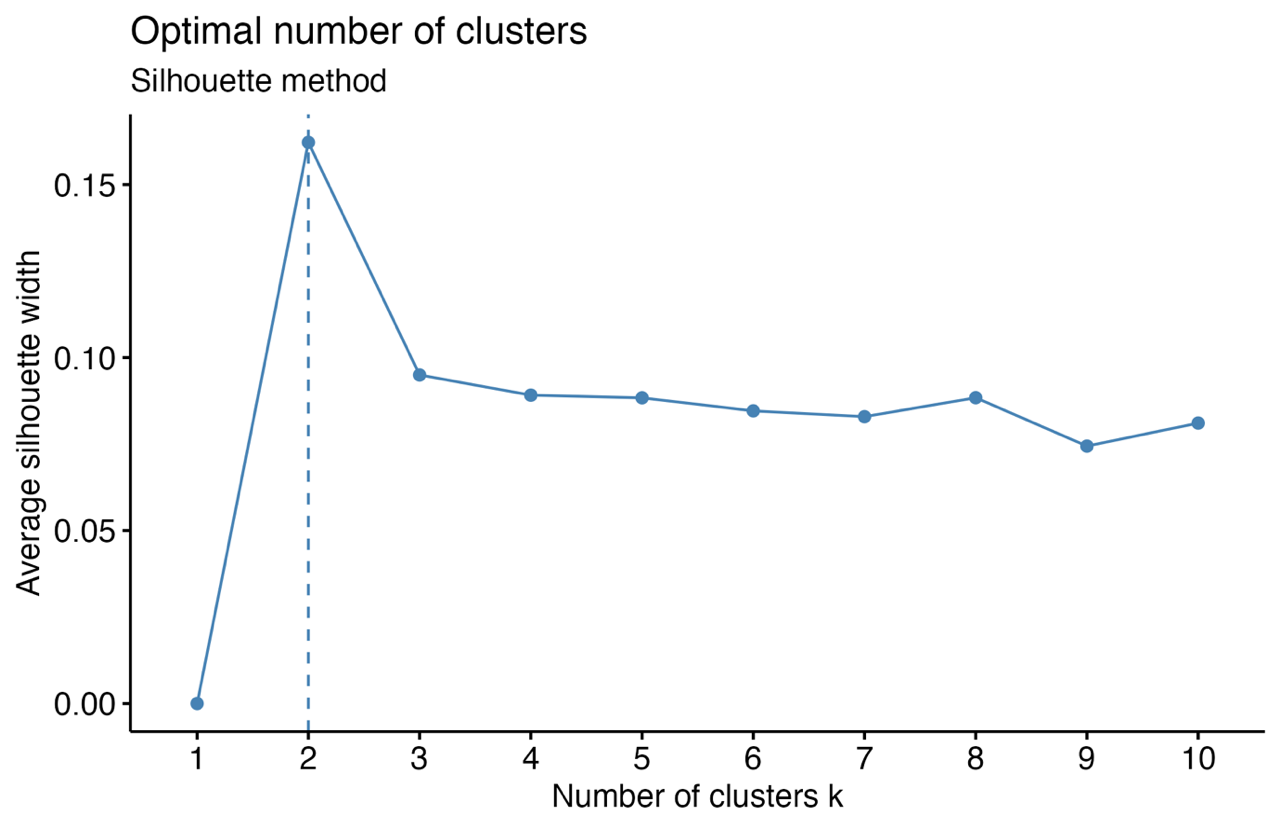


Figure S1. Elbow criterion of silhouette scores for k-means clustering solutions of cognition data over k values 1-10.


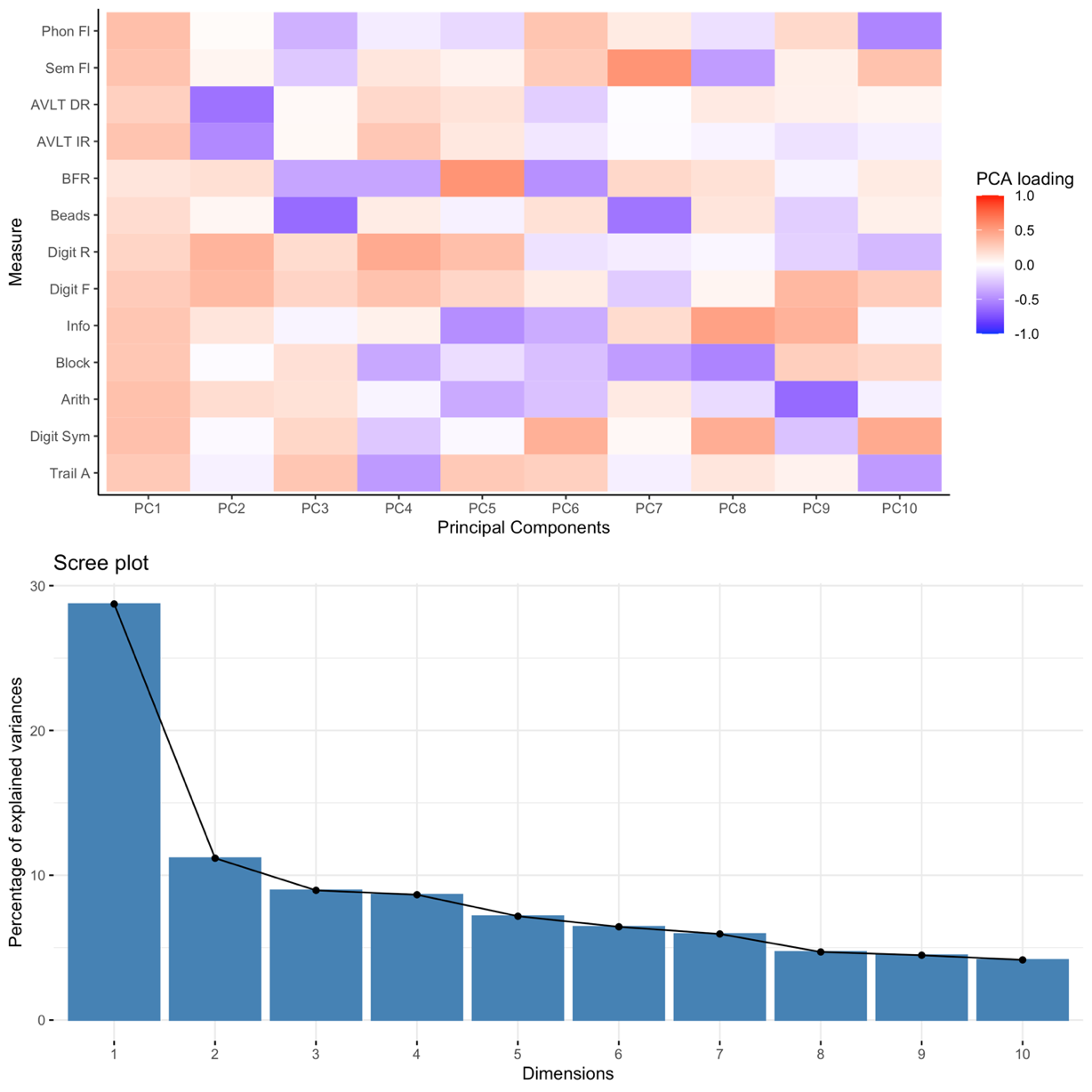


Figure S2. Upper panel: Heat map showing loadings of cognition measures for principal components 1-10 from Principal Component Analysis computed on cognitive data. Lower panel: Scree plot showing the percentage of explained variance for principal components 1-10.


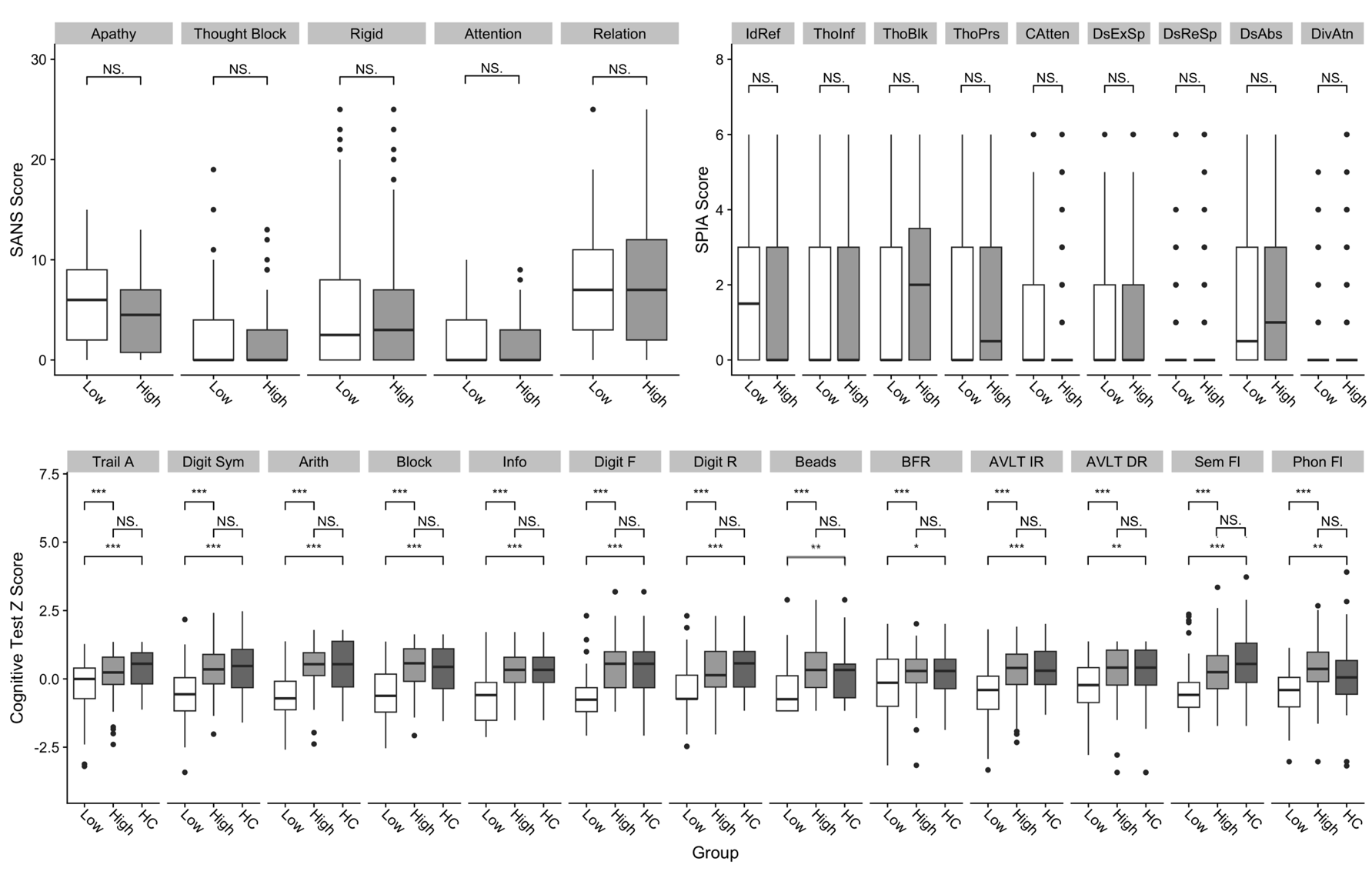


Figure S3. Cognition scores between high / low CHR-P cognition groups defined using a median split of a cognition composite score, and healthy control samples (* pFDR ≤ 0.05, ** pFDR ≤ 0.01, *** pFDR ≤ 0.001). Trail A = Trail making task part A. Digit Sym = Digit symbol, Arith = arithmetic, Block = Block design, Info = Information, Digit F = Digit span task forward, Digit R = Digit span task reverse, Beads = Beads jumping to conclusions task, BFR = Benton Facial Recognition Task, AVLT IR / DR auditory verbal learning task immediate recall / delayed recall, Sem Fl = Verbal learning semantic fluency, Phon Fl = Verbal learning phonemic fluency.

Table S2. Demographics (mean age, sex, mean years of education), mean estimated IQ, mean CAARMS positive / negative summary scores, and medication use for high / low cognition clusters.

|  | High Cognition | Low Cognition | T / X stat | P value |
| --- | --- | --- | --- | --- |
| N | 151 | 140 |  |  |
| Age | 22.66 (4.88) | 22.5 (5.11) | 0.27 | 0.791 |
| Sex (male / female) | 71 (50.71%) / 69 (49.29%) | 86 (56.95%) / 65 (43.05%) | 0.90 | 0.342 |
| Years of Education (SD) | 14.54 (2.86) | 14.15 (3.34) | 1.04 | 0.301 |
| Estimated IQ (SD) | 109.16 (12.71) | 86.35 (11.75) | 15.72 | <0.001 |
| GAF Symptom (SD) | 55.01 (9.39) | 54.46 (11.22) | 0.45 | 0.655 |
| GAF Disability (SD) | 55.62 (12.19) | 54.88 (12.62) | 0.51 | 0.610 |
| CAARMS positive (SD) | 2.46 (1.03) | 2.56 (0.98) | -0.84 | 0.404 |
| CAARMS negative (SD) | 1.97 (0.95) | 2.00 (0.99) | -0.23 | 0.815 |
| Antidepressants | 42 (30.88%) | 38 (29.01%) | 0.04 | 0.841 |
| Antipsychotics | 10 (7.19%) | 16 (12.12%) | 1.37 | 0.242 |
| Transition rate | 26 (17.22%) | 30 (21.43%) | 0.58 | 0.446 |

*Table S3. Demographics (mean age, sex, mean years of education), mean estimated IQ, mean baseline / follow-up Global Assessment of Functioning (GAF), transitioned to psychosis, mean days to follow up, mean CAARMS positive / negative summary scores, and baseline medication use for the Clinical High Risk for Psychosis (CHR) and Healthy Control (HC) samples.*

|  | sMRI data available | sMRI data not available | T / X stat | P value |
| --- | --- | --- | --- | --- |
| N | 194 | 97 |  |  |
| Age | 22.8 (4.78) | 22.13 (5.37) | 1.04 | 0.300 |
| Sex (male / female) | 100 (51.55%) / 94 (48.45%) | 57 (58.76%) / 40 (41.24%) | 1.08 | 0.299 |
| Years Education | 14.51 (3.07) | 14.05 (3.15) | 1.14 | 0.256 |
| Estimated IQ | 97.86 (14.82) | 99 (20.19) | -0.48 | 0.630 |
| Basic Symptom Criteria Met | 100 (51.55%) | 46 (47.42%) | 1.04 | 0.308 |
| Genetic Vulnerability | 32 (16.49%) | 12 (12.37%) | 0.64 | 0.425 |
| Attenuated Symptoms | 161 (82.99%) | 80 (82.47%) | 0.00 | 1.000 |
| BLIP | 14 (7.22%) | 9 (9.28%) | 0.14 | 0.710 |
| GAF Symptom | 53.72 (10.16) | 56.71 (10.33) | -2.31 | 0.022 |
| GAF Disability | 54.08 (12.18) | 57.62 (12.52) | -2.28 | 0.023 |
| GAF Symptom 2 years | 59.83 (14.11) | 60.85 (12.35) | -0.49 | 0.626 |
| GAF Disability 2 years | 62.04 (15.16) | 61.61 (12.87) | 0.20 | 0.843 |
| Transition | 43 (22.16%) | 13 (13.4%) | 2.66 | 0.103 |
| Follow up | 629.48 (253.05) | 675.61 (260.63) | -1.07 | 0.287 |
| CAARMS positive mean | 2.49 (1.05) | 2.54 (0.92) | -0.36 | 0.723 |
| CAARMS negative mean | 2.04 (0.97) | 1.87 (0.95) | 1.38 | 0.170 |
| Antidepressant | 61 (31.44%) | 19 (19.59%) | 2.16 | 0.142 |
| Antipsychotic | 16 (8.25%) | 10 (10.31%) | 0.47 | 0.492 |


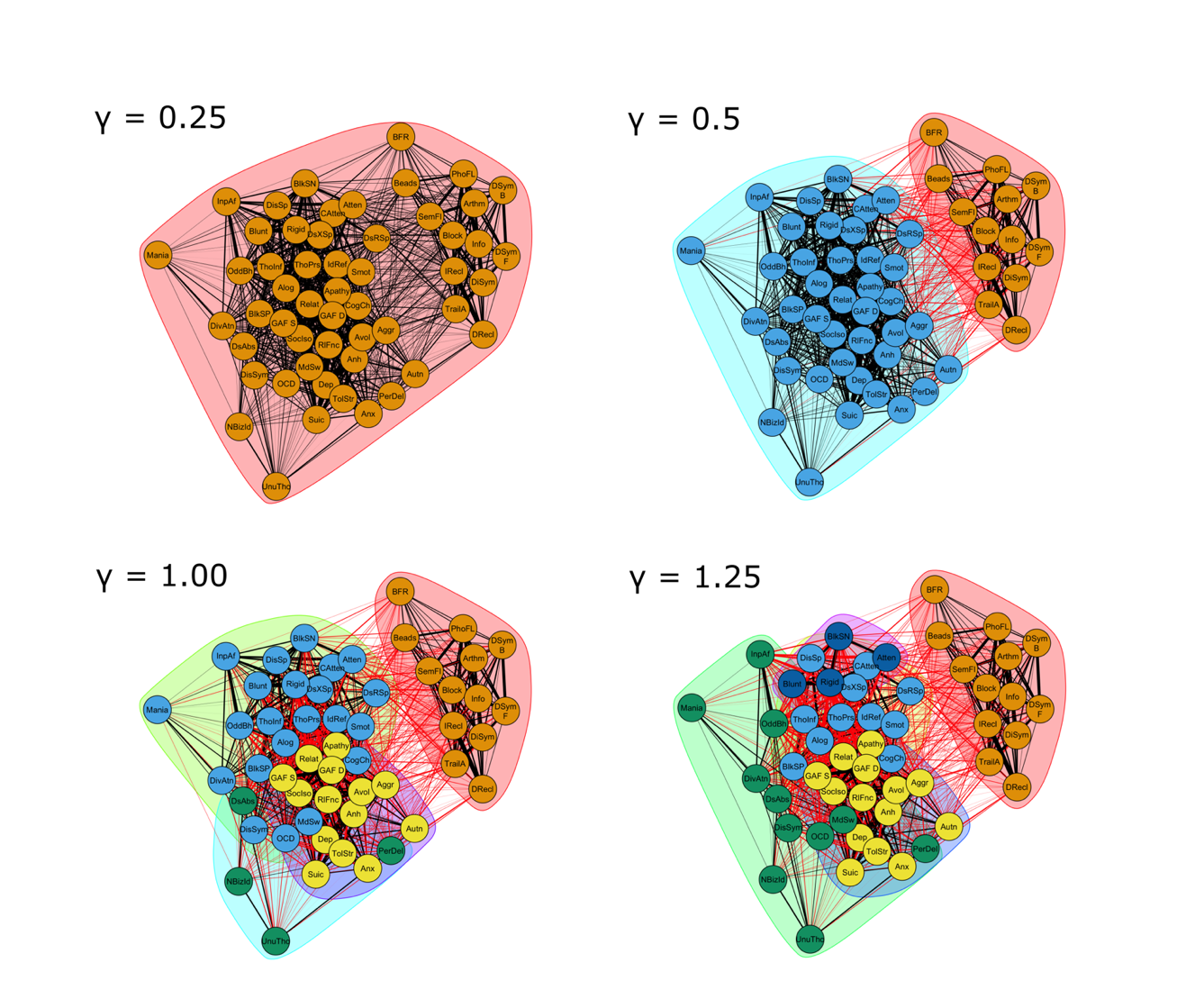


*Figure S4. Weighted network of Spearman’s correlations between cognition, functioning, and symptom scores. Nodes with a degree < 1 were removed. Colours indicate communities of nodes defined using a Louvain community detection algorithm (across resolution parameters γ = 0.25, 0.5, 1.0, 1.25). Black edges show within community connections, red edges show between community connections.*

Cognition: TrailA = Trail Making Task, DiSym = Digit Symbol, Arthm = Arithmetic, Block = Block Design, Info = Information, DS_F = Digit Span Forward, DS_B = Digit Span Reverse, Beads = Beads Task, BFR = Benton Facial Recognition, IRecl = AVLT Immediate Recall, DRecl = AVLT Delayed Recall, SemFl = Semantic Fluency, PhoFL = Phonemic Fluence. Schizophrenia Proneness Instrument-Adult version (SPI-A): IdRef = Ideas Reference, ThoInf = Thought Interferences, BlkSP = Thought Block, ThoPrs = Thought Pressure, CAtten = Captivation Attention, DsXSp = Disturbance Expressive Speech, DsRSp = Disturbance Receptive Speech, DsAbs = Disturbance Abstract Thinking, DivAtn = Inability Divide Attention. Scale for the Assessment of Negative Symptoms: Apathy = Apathy, BlkSN = Thought Block, Rigid = Rigidity, Atten = Attention, Relat = Relation. Comprehensive Assessment of At Risk Mental States: Dep = Depression, Suic = Suicidality, Anh = Anhedonia, Avol = Avolition, Mania = Mania, MdSw = Mood Swing, Aggr = Aggression, UnuTho = Unusual Thought, NBizId = Non-bizzare Ideas, PerDel = Perceptual Abnormalities, DisSp = Disorganised Speech, Anx = Anxiety, OCD = Obsessive Compulsive Disorder, DisSym = Disorganised Symptoms, TolStr = Tolerance to Everyday Stress, CogCh = Cognitive Change, Alog = Alogia, Blunt = Blunted Affect, InpAf = Inappropriate Affect, SocIso = Social Isolation, RlFnc = Role Functioning, OddBh = Odd Behaviour, Smot = Subjective Motor Change, Autn = Autonomic Functioning
